# Lovastatin production by an oleaginous fungus, *Aspergillus terreus* KPR12 using sago processing wastewater (SWW)

**DOI:** 10.1186/s12934-022-01751-2

**Published:** 2022-02-14

**Authors:** Naganandhini Srinivasan, Kiruthika Thangavelu, Sivakumar Uthandi

**Affiliations:** 1 Biocatalysts Laboratory, Department of Agricultural Microbiology, Tamil Nadu Agricultural University, Coimbatore, Tamil Nadu 641 003 India; 2 Department of Renewable Energy Engineering, Agricultural Engineering College and Research Institute, Tamil Nadu Agricultural University, Coimbatore, Tamil Nadu 641 003 India; 3 Present Address: Department of Agriculture Engineering, Mahendra Engineering College, Namakkal, Tamil Nadu 637 503 India

**Keywords:** *Aspergillus*, Lovastatin, Sago wastewater, Bioassay

## Abstract

**Background:**

Lovastatin is one of the first statins to be extensively used for its cholesterol-lowering ability. It is commercially produced by fermentation. Species belonging to the genus *Aspergillus* are well-studied fungi that have been widely used for lovastatin production. In the present study, we produced lovastatin from sago processing wastewater (SWW) under submerged fermentation using oleaginous fungal strains, *A. terreus* KPR12 and *A. caespitosus* ASEF14.

**Results:**

The intra- and extracellular concentrations of lovastatin produced by *A. terreus* KPR12 and *A. caespitosus* ASEF14 were lactonized. Because *A. caespitosus* ASEF14 produced a negligible amount of lovastatin, further kinetics of lovastatin production in SWW was studied using the KPR12 strain for 9 days. Lovastatin concentrations in the intra- and extracellular fractions of the *A. terreus* KPR12 cultured in a synthetic medium (SM) were 117.93 and 883.28 mg L^–1^, respectively. However, these concentrations in SWW were 142.23 and 429.98 mg L^–1^, respectively. The yeast growth inhibition bioassay confirmed the antifungal property of fungal extracts. *A. terreus* KPR12 showed a higher inhibition zone of 14 mm than the ASEF14 strain. The two-way analysis of variance (ANOVA; *p* < *0.01*) showed significant differences in the localization pattern, fungal strains, growth medium, and their respective interactions. The lovastatin yield coefficient values were 0.153 g g^–1^ on biomass (Y_LOV/X_) and 0.043 g g^–1^ on the substrate, starch (Y_LOV/S_). The pollutant level of treated SWW exhibited a reduction in total solids (TS, 59%), total dissolved solids (TDS, 68%), biological oxygen demand (BOD, 79.5%), chemical oxygen demand (COD, 57.1%), phosphate (88%), cyanide (65.4%), and void of nutrients such as nitrate (100%), and ammonia (100%).

**Conclusion:**

The starch-rich wastewater serves as a suitable medium for *A. terreus* KPR12 for the production of lovastatin. It simultaneously decontaminates the sago processing wastewater, enabling its reuse for irrigation/recreation.

**Graphical Abstract:**

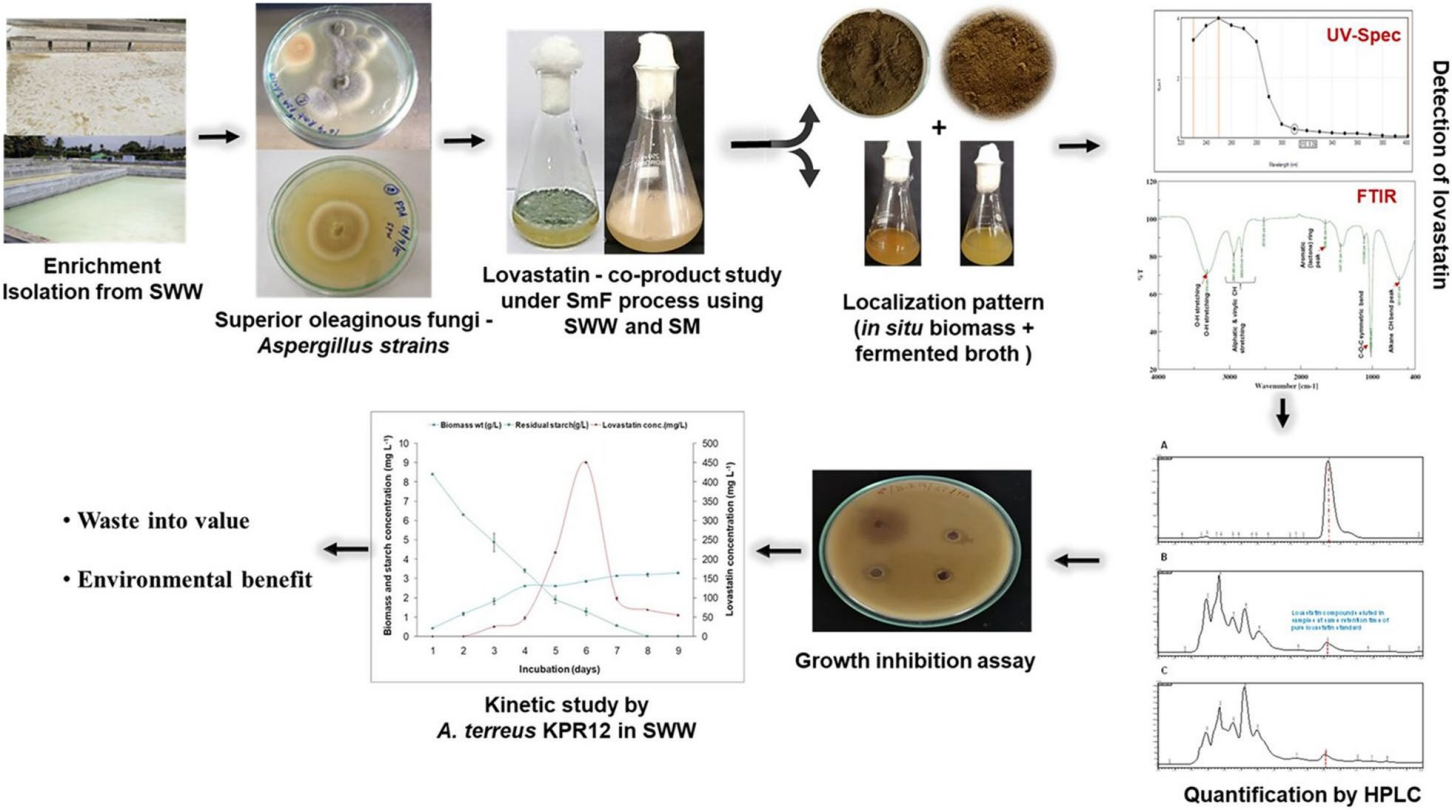

## Background

Hypercholesterolemia is a well-studied metabolic disorder associated with cardiovascular morbidity and mortality in human adults [1]. Statins are widely used as cholesterol-lowering drugs that hinder the activity of the critical catalyst, 3-hydroxy-3-methylglutaryl coenzyme A (HMG-CoA) reductase (mevalonate: NADP1 enzyme EC 1.1.1.34), which is involved in the endogenous biosynthesis of LDL cholesterol [2, 3]. Among statins, lovastatin is the first drug approved by the US Food and Drug Administration (FDA) in 1987 for the treatment of hypercholesterolemia [4]. Lovastatin has been reported to possess anticancer properties, immunomodulatory function, and anti-inflammatory activity. In addition, it is known to play a significant role in preventing neurological disorders and bone problems [5–7]. Lovastatin is a fungal secondary metabolite produced through the polyketide pathway. Several fungal genera such as *Aspergillus*, *Penicillium*, *Monascus*, *Paecilomyces*, *Trichoderma*, *Scopulariopsis*, *Doratomyces*, *Phoma*, *Pythium*, *Gymnoascus*, *Hypomyces*, and *Pleurotus* are known as lovastatin producers [8–12]. Of which, *Monascus ruber* and *Aspergillus terreus* are the foremost and targeted industrial producers of lovastatin [4, 13].

Lovastatin is produced using different fermentation strategies, including surface fermentation, solid-state fermentation (SSF), and submerged fermentation (SmF) [14, 15]. For large-scale commercial production, SmF is used in batch and fed-batch modes [15]. A rich nutrient broth could be used for the production of lovastatin in the SmF process. Although several agro-wastes are used as substrates in the SSF process owing to their low cost, eco-safety, long-term availability, and easy downstream processing [16], no research has been conducted on the use of industrial wastewater.

India is one of the world’s largest producers of cassava, which results in a wastewater discharge of about 40,000 to 50,000 L and 15 to 30 tons of sludge per unit per day to produce flour and starch [17, 18]. Sago processing industries produce two types of wastewaters. The first type is released by the washing and peeling of cassava tubers and has low chemical oxygen demand (COD). The second type is released during the extraction of starch; it has a high pollution load due to a high COD and biological or biochemical oxygen demand (BOD); contains starch up to 7% [19] and low concentrations of cytotoxic compounds or growth inhibitors [20]. The reported starch content of SWW was 4.82 g L^−1^ [21].

Applications of SWW include biogas [22, 23], hydrogen [24, 25], microbial lipid and biodiesel production using oleaginous yeast and fungi [21, 26–29]. Several oleaginous fungi and yeasts were isolated previously from this wastewater for biodiesel production with simultaneous removal of pollutants [27–31]. Furthermore, certain hyper oleaginous fungi such as *A. terreus* KPR12 and *A. caespitosus* ASEF14 accumulate more than 20% of their dry weight lipid [26].

We produced lovastatin with a high therapeutic value using these known lovastatin-producing fungal strains and low-cost or zero-cost waste stream sago processing wastewater (SWW) and simultaneously performed its decontamination. The produced lovastatin in SWW was characterized and quantified using ultraviolet (UV) spectrometry, Fourier transform infrared (FTIR) spectroscopy, and high-performance liquid chromatography (HPLC). The lovastatin biogenesis of *A. terreus* KPR12 in SWW was explained through a simple kinetic model.

To the best of our knowledge, this is the first report on lovastatin production using SWW. This study indicates the prospect of exploiting cheaper, large, and underutilized industrial effluent as a potential resource for the production of lovastatin in addition to the sequestration of hazardous pollutants present in SWW.

## Materials and methods

### Fungal strains and culture conditions

*A. caespitosus* ASEF14 and *A. terreus* KPR12 were isolated, identified, characterized, and screened for oleaginicity, amylase secretion, and cyanide degradation in SWW [27, 28, 30]. In addition to biolipid production, these two fungal strains were screened for the production of co-metabolite, lovastatin in synthetic medium (SM), as well as SWW [26]. The GenBank accession numbers of these strains are MF599090 and MF599091. The cultures were maintained on potato dextrose agar (PDA) slants at 4 °C.

### Physicochemical characterization of SWW

The collection and characterization of SWW used in the present work have been reported in our previous work [28]. The initial starch concentration of SWW was adjusted to 10 g L^−1^, and other physicochemical parameters included pH 4.6, electrical conductivity (EC) 6.3 dS m^−1^, salinity 4.86 g L^–1^, total solids (TS) 4.57 g L^–1^, total dissolved solids (TDS) 4.16 g L^−1^, nitrate 3.10 mg L^−1^, ammonia 5.48 mg L^−1^, phosphate 611.67 mg L^−1^, biological oxygen demand (BOD) 5.04 g L^−1^, chemical oxygen demand (COD) 70.67 g L^−1^, and cyanide 4.46 mg L^−1^.

### Preparation of seed inoculant

The fungal strains of *A. terreus* KPR12 and *A. caespitosus* ASEF14 were grown on PDA incubated at 30 °C for 5 days, and stored under refrigeration at 4 °C. The conidiospores from the above strains were harvested separately with sterile solution (0.05% Tween 80 and 0.9% NaCl), washed twice with 0.1 M sterile phosphate buffer (pH 6), and adjusted to contain 10^7^ spores mL^–1^. An aliquot of a spore suspension of each culture (1 mL) was inoculated into 50 mL of potato dextrose broth (pH 6.5) in a 250 mL Erlenmeyer flask and incubated at 30 °C under a static condition for 72 h until the exponential growth was reached.

### Fermentation conditions and lovastatin production

The two fungal strains grown under SmF conditions in SM and SWW were tested for lovastatin production. About 100 mL of sterile SM and SWW were taken in a 250 mL Erlenmeyer flask, and 10% of prepared liquid seed inoculum of *A. caespitosus* ASEF14 and *A. terreus* KPR12 was inoculated separately to the production media in the flasks. Before inoculation, the pH of both liquid substrates was adjusted to 6.5 using 0.1 N HCl or 0.1 N NaOH. The initial starch content of SWW was 4.82 g L^−1^ and adjusted to 10 g L^−1^. The flasks were incubated at 30 °C for 6 days under non-shaking conditions. The composition of the SM media (per L) was as follows: 10 g starch, 0.5 g ammonium sulfate, 7 g potassium dihydrogen phosphate, 2.5 g disodium hydrogen phosphate, 1.5 g magnesium sulfate, 0.15 g ferric chloride, 0.15 g calcium chloride, 0.02 g zinc sulfate, and 0.06 g manganese sulfate.

### Biomass estimation

After fermentation, fungal mats in SM and SWW were separated by filtration through pre-weighed Whatman grade 1 filter paper. The biomass obtained by filtration was washed twice with distilled water and subjected to drying at 50 °C until it reached a constant weight. The dry weight of biomass was calculated by gravimetric analysis [12].

### Extraction of intracellular lovastatin

To measure the intracellular concentrations of statin, the dry mycelium (0.5 g) was ruptured by ultrasonication for 5 min (PCI Analytics; Mumbai, India). The sonicated samples were adjusted to pH 3.0 using 2 NH_3_PO_4_ and extracted with 10 mL of ethyl acetate in a shaker incubator at 180 rpm at 30 °C for 2 h. The organic and aqueous phases of the filtrates were separated by cold centrifugation (4 °C) at 6000 rpm for 10 min. The organic phases were collected, lactonized with 1% trifluoroacetic acid, and concentrated under reduced pressure. The dried residue was dissolved in 1 mL acetonitrile, filtered through a 0.45 µm filter, collected in clean brown glass vials, and stored at 4 °C for ultraviolet (UV) spectrophotometry, Fourier transform infrared (FTIR) spectroscopy, and high-performance liquid chromatography (HPLC) analysis [32].

### Extraction of extracellular lovastatin

To measure the extracellular concentrations of lovastatin, the fermentation broths of SM and SWW were acidified to pH 3.0 by the addition of 10% 1 N HCl. The acidified broths were extracted with an equal volume of ethyl acetate under shaking conditions (180 rpm) at 30 °C for 2 h. The organic and aqueous phases of filtrates were separated by cold centrifugation (4 °C) at 6000 rpm for 10 min. The organic phases were collected, lactonized, concentrated, and analyzed as intracellular lovastatin [32].

### Analytical methods

#### UV spectrophotometric method

The filtered fungal extracts were analyzed qualitatively for the presence of lovastatin using UV–visible spectrophotometer (SpectraMax i3x, Sunnyvale, California, US) [33]. The radiation source was a deuterium lamp emitting a continuous UV spectrum between 210 and 360 nm. The lovastatin spectrum was recorded in the absorbance mode at 247 nm and 258 nm, respectively. Pure lovastatin (Sigma Aldrich, St. Louis, Missouri, US) was used as a standard for comparison.

#### Fourier transform-infrared spectroscopy

FTIR measurements of the samples were performed using attenuated total reflectance (ATR) equipped with a deuterated triglycine sulfate (DTGS) detector (JASCO FT/IR-6300, Japan). The crude sample (10 µL) was directly placed on the surface of the diamond crystal. Samples were scanned using absorbance spectra at wavenumbers 400 to 4000 cm^−1^ at a resolution of 1 cm^−1^ for each interferogram.

#### High-performance liquid chromatography

The sample extracts were quantitatively analyzed for the presence of lovastatin using HPLC device, Shimadzu Nexera X2 (Shimadzu, Prominence HPLC, Kyoto, Japan) with a UV detector and a C18 column. Acetonitrile and water (acidified with 1.1% phosphoric acid) in the ratio of 70:30 v/v were used as mobile phase. The eluent flow rate and the column temperature were maintained at 1 mL min^–1^ and 40 °C, respectively. The detection was performed at 238 nm wavelengths, with an injection volume of 20 µL. Lovastatin standard was prepared according to the manufacturer’s instructions [34]. Lovastatin was identified in the sample by comparing the retention times with the standards.

### Kinetics of lovastatin production in SWW by *A. terreus* KPR12

A 250 mL Erlenmeyer flask containing approximately 100 mL of SWW was sterilized, inoculated with 10% of *A. terreus* KPR12 inoculum, and incubated at 30 °C. The culture broth was harvested from day 1 until day 9 to monitor the growth of strains and production of lovastatin. The cell dry weight was determined by gravimetric analysis. The amount of lovastatin was determined using HPLC as mentioned in the analytical methods section. Residual starch in SWW was analyzed using the phenol sulfuric acid method [35]. The following kinetic and stoichiometric parameters used to describe the growth of strains and production of lovastatin by *A. terreus* KPR12 was determined.

The substrate consumption rate (*r*) is expressed in days.

1 $$r = \left( {S_{i} {-}S_{o} } \right)/\Delta {\text{t,}}$$

where *S*_*i*_ is the initial concentration and *S*_*o*_ is the final concentration of substrate (s).

The lovastatin yield coefficient (Y) was determined relative to the production of biomass (X) or the consumption of total substrate (S) in the reaction.

2 $${\text{Y}}_{{{\text{LOV}}/{\text{X}}}} = \left( {P_{max} {-}P_{i} } \right)/(X_{max} {-}X_{i} )$$

3 $${\text{Y}}_{{{\text{LOV}}/{\text{S}}}} = \left( {P_{max} {-}P_{i} } \right)/(S_{i} {-}S_{o} )$$

P_max_ is the maximum concentration of lovastatin, and P_i_ is the initial concentration of lovastatin in the above equation. µ_max_ is the maximum specific growth rate obtained from a plot of the specific biomass concentration versus time.

### Bioassay

The yeast growth inhibition bioassay was performed using the agar well diffusion method [36]. *Candida tropicalis* ASY2 (Acc no. MH011502) was used as a test organism. Cells of the *C. tropicalis* ASY2 were suspended in phosphate-buffered saline and spread onto the yeast extract peptone dextrose (YEPD) medium. Wells were made using a sterile cork borer of 6 mm diameter. Further, 100 µL of intra- and extracellular extract of the fungus KPR12 was loaded into separate wells. Ethyl acetate and the standard solution of lovastatin (10 mg dissolved in 100 mL of ethyl acetate) (Sigma Aldrich) were used as negative and positive controls, respectively. The standard was prepared according to the method of Friedrich et al. [37] with a slight modification, in which the lovastatin was suspended in ethyl acetate followed by sonication and filtration. All plates were incubated at 30 °C for 16 to 24 h. A clear inhibition zone around the indicator organisms was observed, and the diameter of the inhibition zone is proportional to the concentration of lovastatin in samples.

### Characterization of decontaminated SWW

The nutrient and toxicant removal efficiency of *A. terreus* KPR12 in the SWW was studied along with lovastatin kinetics. After fermentation, the spent SWW was filtered, and the physicochemical parameters were determined according to the standard method of water and wastewater analysis [38]. The cyanide content in SWW was estimated using the modified picric acid method [39].

### Statistical analysis

Data were subjected to statistical analysis using the Microsoft Excel for Windows 2007 add-ins with XLSTAT version 2010.5.05 [40], and all experiments were performed in triplicate. Statistically significant differences between the means of groups and their interactions were determined using one-way and two-way analysis of variance (ANOVA) and Duncan’s multiple range test (DMRT) at the 5% significance level.

### Geolocation information

The Tamil Nadu Agricultural University’s global positioning system (GPS) coordinates are latitude: 11° 07′ 3.36ʺ N and longitude: 76° 59′ 39.91ʺ E.

## Results and discussion

In the present study, we produced cholesterol-reducing lovastatin using SWW under SmF using oleaginous fungal strains *A. caespitosus* ASEF14 and *A. terreus* KPR12. Initially, the fermentation was performed for 6 days. After the extraction of lovastatin from the broth of SM and SWW (extracellular) and fungal mycelium (intracellular), it was acidified and lactonized with 1% trifluoroacetic acid. This process can transform the acid form of lovastatin into the lactone form.

**Fig. 1:**
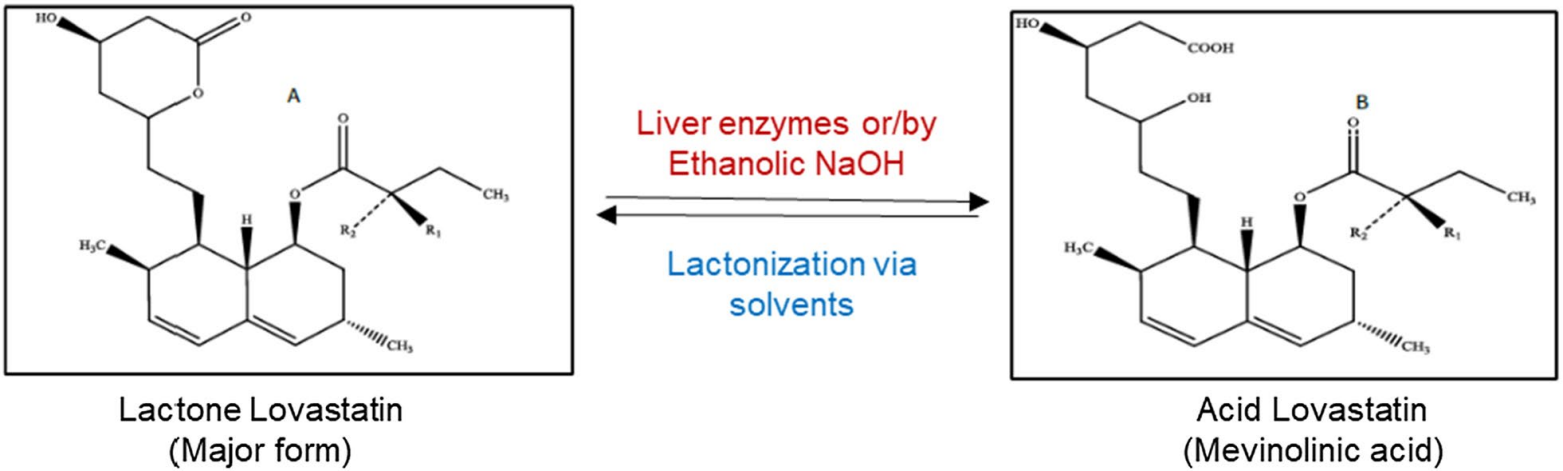
Closed-ring lactone (inactive) and open-ring hydroxy form (active) of lovastatin produced by filamentous fungi

Generally, lovastatin exists in both open-ring β-hydroxy acid (active) and closed-ring β-lactone forms (inactive) (Fig. 1). The physicochemical and pharmaceutical properties of these two forms are different and interchangeable [6, 41]. In the broth culture media, the filamentous fungi secrete lovastatin mostly in its hydroxy acid form. However, the lactone form of industrial lovastatin makes it a viable option for subsequent quantification analyses. Therefore, the reduction in pH and lactonization converts the acid form to lactone for the quantification of lovastatin [13, 41]. In the present investigation, the adopted techniques ensured the accurate quantification of lovastatin in fermentation broth samples.

### Analysis of lovastatin in fungal crude extracts

**Fig. 2:**
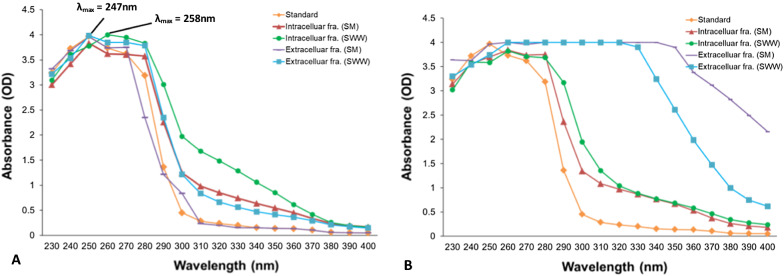
UV spectrophotometric analysis of intra- and extracellular fractions of *A. terreus* KPR12 (**A**) and *A. caespitosus* ASEF14 (**B**) grown in SM and SWW under SmF

The lactonized lovastatin extracts from the samples were qualitatively analyzed using the UV–visible spectrophotometer and compared to the lovastatin absorption spectrum (Fig. 2A). The lovastatin compound had a UV-absorbing peak at 247 nm (Fig. 2A). Such an absorption band corresponds to the π–π transition due to the conjugated double bonds. As seen in Fig. 2A, intra- and extracellular fractions of *A. terreus* KPR12 from SM and SWW had the same UV absorption spectra as the lovastatin standard (λ_max_ = 247, 258 nm). The UV absorption spectra of intra- and extracellular fractions of *A. caespitosus* ASEF14 (Fig. 2B) revealed that the intracellular fraction exhibited an absorption spectrum similar to that of the lovastatin standard. In contrast, the extracellular fraction of SM and SWW revealed a distinct pattern, such as stationary phase lines indicated the presence of non-lovastatin compounds. It has been reported three different maximum absorptions at 232, 238, and 247 nm of pure lovastatin, suggesting its better identification from other compounds, which is due to the presence of dienes [6, 42]. The spectrophotometric analysis of lovastatin is easy, quick, eco-friendly, and less laborious than other analytical techniques. Based on these observations, the UV absorption spectrum of *A. terreus* KPR12 confirmed the synthesis of lovastatin.

### FTIR spectral analysis of lovastatin

**Fig. 3:**
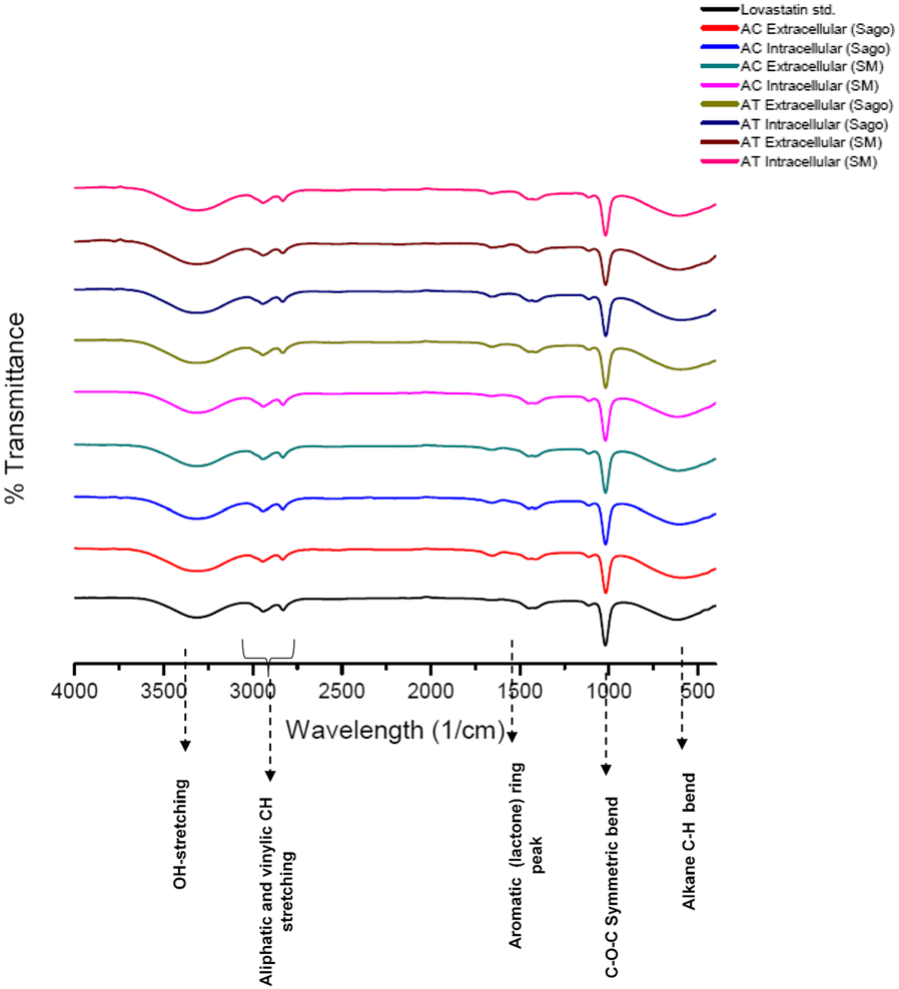
Functional groups corresponding to lovastatin identified in intracellular and extracellular fractions of *A. terreus* KPR12 and *A. caespitosus* ASEF14 in SM and SWW by FTIR spectral analysis and compared with the pure lovastatin standard

The FTIR spectra of fungal extracts were analyzed by interferometry using the pure lovastatin standard (Fig. 3). All spectra were recorded in the range of 400 to 4000 cm^−1^. A narrow band at 3400 to 3500 cm^−1^ indicated the presence of non-hydrogen bonded O–H stretches. Vibration often occurs to the left of this peak, suggesting the alcoholic/phenolic hydroxyl groups. The olefinic C–H stretching vibration band observed at 2941.88 cm^−1^ is a particular characteristic of chitin, a crucial component of the cell wall, and ergosterol [43]. The peaks between 2900 and 3000 cm^−1^ are aliphatic and vinylic C–H stretching. Similarly, a band at 1447.31 cm^−1^ represented two carbonyl ester groups for bending vibrations in methyl and methylene groups. The symmetric bending of the C–O–C ester and alkane C–H bonds at 1020.16 cm^−1^ and between 680 and 610 cm^−1^, respectively, corresponds to specific functional peaks of lovastatin (Fig. 3). The C–H stretching absorptions were observed below 3000 cm^−1^. Certain band structures observed between 3150 and 3000 cm^−1^ represents unsaturation (C=C–H) and aromatic rings. The other most important bands were aromatic ring vibrations at around 1500 to 1600 cm^−1^, which usually appeared as a pair of band structures in the lovastatin [44]. These FTIR spectra confirmed the presence of lovastatin in the fungal extracts and fractions (Fig. 3).

### Quantification of lovastatin

**Fig. 4:**
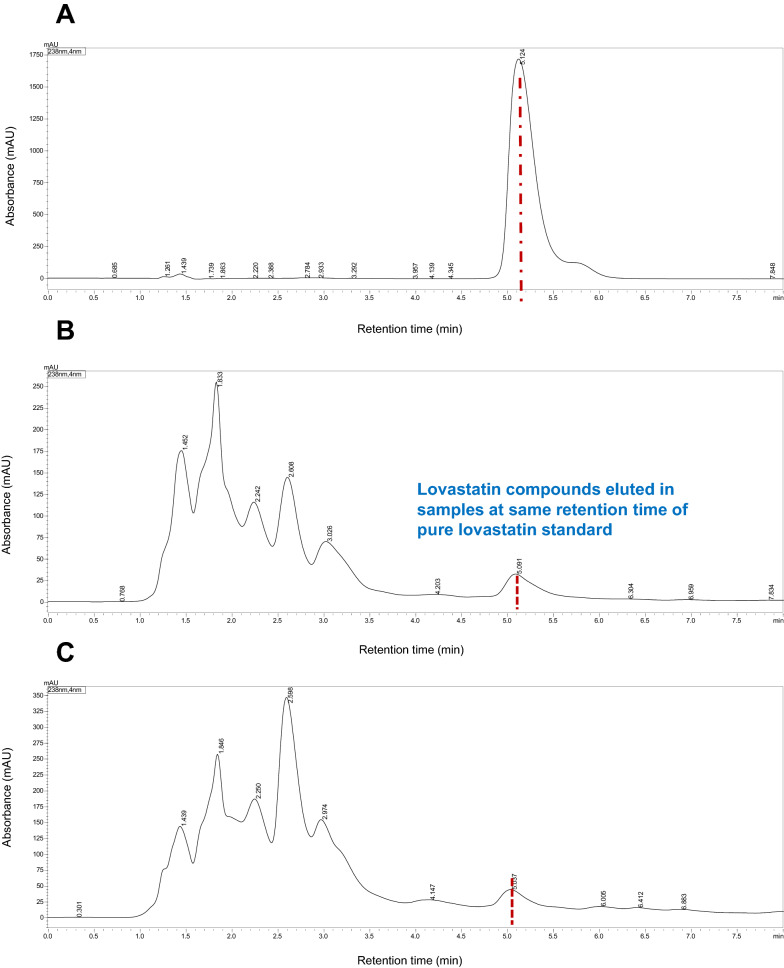
HPLC chromatograms of pure lovastatin standard (**A**), fermented extract of *A. terreus* KPR12 (**B**), and *A. caespitosus* ASEF14 (**C**)

**Fig. 5:**
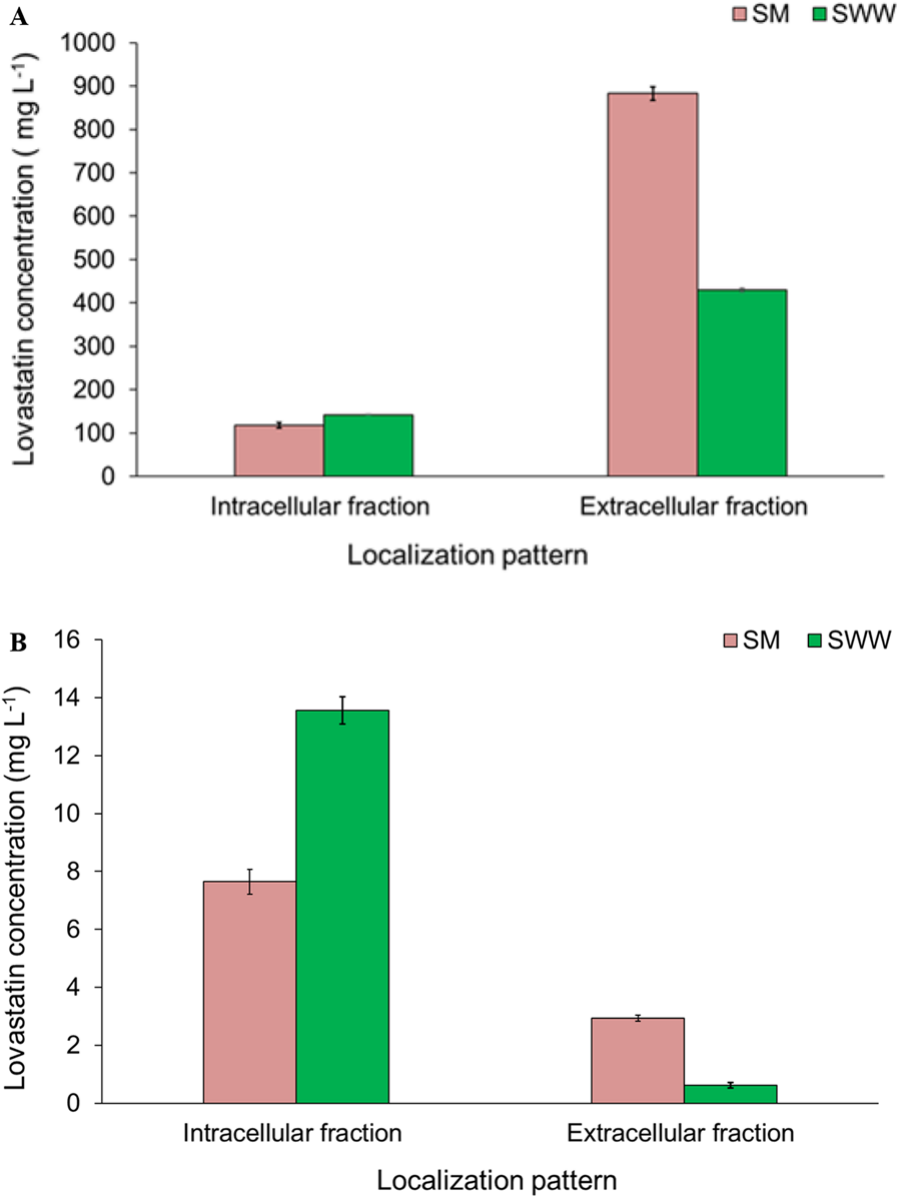
Lovastatin content in intracellular and extracellular fractions of *A. terreus* KPR12 (**A**) and *A. caespitosus* ASEF14 (**B**) grown in SM and SWW

Lovastatin produced by two different fungal strains grown in SM and SWW was quantified using HPLC. The retention time (5.124) of the first peak for both fungal extracts was similar to the standard lovastatin, and the appearance of other peaks in the samples might be due to the presence of impurities or unidentified compounds produced during the fermentation process (Fig. 4A–C). The concentrations of lovastatin in the intra- and extracellular fractions of KPR12 grown in the SM were 117.93 and 883.28 mg L^−1^, respectively; however, in SWW, lovastatin yield were 142.23 and 429.98 mg L^−1^, respectively (Fig. 5A). Moreover, the lovastatin concentrations in intra- and extracellular fractions of ASEF14 grown in SM were 7.64 and 2.94 mg L^−1^, and in SWW, these were 13.57 and 0.62 mg L^−1^, respectively (Fig. 5B). The results demonstrated that the fungal strain KPR12 was superior to ASEF14 in terms of intra- and extracellular fractions, irrespective of SM and SWW. Therefore, a further experimental study focused only on the high lovastatin-yielding fungus KPR12.

The media conditions and compositions exert varying effects on the production of fungal secondary metabolites [45]. The synthesis of such secondary metabolites occurs at the end of the logarithmic (log) growth phase, in which the essential nutrients are in low supply. The secretion of accumulated metabolites into the surrounding medium is necessary. Fungal strains grown on a synthetic starch-based substrate medium can secrete a high amount of lovastatin as an extracellular fraction. In this study, a 2.04 fold increase and 1.2 fold decrease was observed in extracellular and intracellular concentrations of lovastatin by KPR12 in the SM compared to SWW, respectively. This lower secretion could be attributed to the mass transfer resistance limits in SWW [46]. In addition, SWW contains hydrogen cyanide (HCN), which is generated during milling processes such as peeling, slicing, squeezing, and crushing cassava tubers. At high concentrations, cyanide becomes toxic to living organisms. Apart from its toxic nature, cyanide is well-known for its metabolic inhibitory effects [47, 48]. This may also affect the extracellular secretion by fungi. Ultimately, the extraction of lovastatin from the intracellular portion of fungal cultures complicates the downstream processing due to the presence of structural analogs and intermediates [49]. The production of lovastatin is highly influenced by slowly metabolized carbon sources (lactose, glycerol, and fructose) compared to glucose [8, 50]. The pathway leading to lovastatin synthesis using carbon is slower than the one that uses carbon for biomass production (glucose) because lovastatin is a product of secondary metabolism. Thus, starch, a slowly metabolized carbon source present in SWW and SM, could affect the production of biomass and lovastatin.

Although the lovastatin content was lower in SWW than in SM; it was selected for kinetic analysis owing to its low-cost nature, high availability, economic factors, and environmental impact. All data were analyzed using a two-way ANOVA, and the results indicated significant differences (*p* < *0.05*) in the localization pattern, fungal strains, growth media, and their interactions (Table 1).

**Table 1:** Statistical parameters of two-factor ANOVA of lovastatin production as affected by cultivation medium, strains, and localization

Effect	SS	DF	MS	*F*	Prob F	Sign
Growth medium	45,240.80	1	45,240.80	607.68	7.8354 × 10^–9^	**
Strains	599,563.59	1	599,563.59	8053.44	2.65301 × 10^–13^	**
Fraction	268,032.72	1	268,032.72	3600.26	6.61319 × 10^–12^	**
Growth medium × strains	46,782.23	1	46,782.23	628.39	6.86322 × 10^–9^	**
Growth medium × fraction	59,010.85	1	59,010.85	792.64	2.73655 × 10^–9^	**
Strains × fraction	286,637.61	1	286,637.61	3850.17	5.05888 × 10^–12^	**
Growth medium × strains × fraction	55,069.78	1	55,069.78	739.71	3.59881 × 10^–9^	**
Residual	595.58	8	74.45			
Total	1,360,933.16	15	90,728.88			
CV (%)	4.32					

Growth medium—SM and SWW; Strains—*A. terreus* KPR12 and *A. caespitosus* ASEF14; Fraction—Intracellular and extracellular fraction; SS—sum of the squares; DF—degrees of freedom; MS—Mean sum of the squares; *F*—F test; Prob F—F probability; Sign.—significant at *p < *0.05*; **p < *0.01*

### Kinetics of lovastatin production by *A. terreus* KPR12 in SWW

**Fig. 6:**
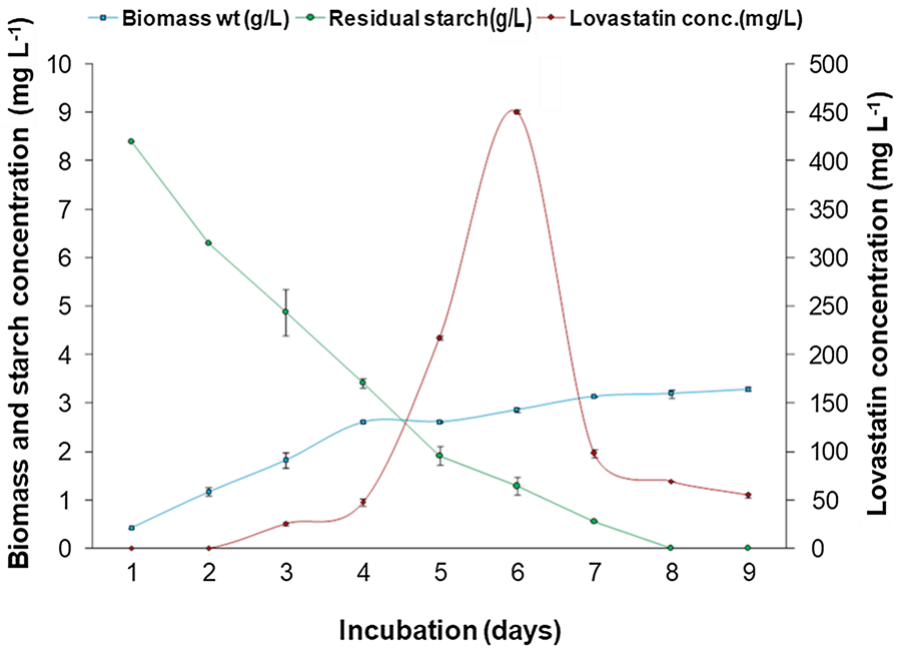
Growth kinetics and lovastatin production by *A. terreus* KPR12 in SWW

The fermentation cycle was conducted for 9 days. The lovastatin content, dry cell weight, residual starch, and other physicochemical changes were measured periodically in SWW (Fig. 6). The results revealed that lovastatin was not detected in the first 2 days of fermentation. Lovastatin, a product of secondary metabolism, is produced at the end of the log or during stationary growth phase of fungi. It cannot secrete or synthesize at the early growth stage of fungi [15]. The secretion of lovastatin in SWW started on the third day of fermentation using 5.13 g L^−1^ starch and produced biomass of 1.82 g L^−1^. The maximum extracellular concentration of lovastatin was 451 mg L^−1^ with a dry weight of 2.86 g L^−1^ on the 6th day of fermentation.

The lovastatin synthesis pathway consumes carbon more slowly than the biomass growth process. The synthesis of building blocks for biomass synthesis is hindered by nitrogen limitation, and the extra carbon is channeled into lovastatin production. Polyketide synthase (PKS: non-aketide synthase [LNKS] + diketide synthase [LDKS]), a multifunctional enzyme complex, is involved in the biosynthesis of lovastatin [51]. This enzyme followed the hyperbolic relationship when the substrate concentration was low. In the current investigation, a steep increase in the rate of reaction (lovastatin synthesis) with the availability of substrate was observed, i.e., starch (Fig. 6). When starch is unavailable, the enzyme catalytic site becomes vacant [52]. Thus, the rate at which lovastatin synthesis drops dramatically (Fig. 6).

The results showed that the lovastatin yield would be enhanced if essential nutrients were present in the medium. Such findings were consistent with Hajjaj et al. [53] and observed that the relatively low levels of lovastatin produced (0.034 mg g^−1^ h^−1^) in cultures growing at a high specific growth rate (0.070 h^−1^), whereas higher productivity (0.093 mg g^−1^ h^−1^) was achieved at lower growth rates (0.052 h^−1^). Starvation due to a lack of essential nutrients (no residual starch content in SWW) in this study appeared to block fungal growth and lovastatin production.

The kinetic parameters of *A. terreus* KPR12 grown in SWW are shown in Table 2. The adjusted initial starch content used for this kinetic study was 10 g L^−1^. The final biomass (X_FINAL_) (on a dry weight basis) obtained by the fungi in SWW was 3.20 g L^−1^. The lovastatin yield coefficients on biomass (Y_LOV/X_) and on the substrate (Y_LOV/S_) were found to be 0.153 and 0.043 g g^−1^, respectively. Bizukojc and Ledakowicz [54] documented lovastatin yield coefficients of 0.0065 and 0.0050 g g^−1^ by *A. terreus* using lactose and glycerol in the culture, respectively. Lovastatin to biomass yield coefficient was 0.0052, and the initial lactose and glycerol contents were 10 and 5 g L^−1^, respectively. The results showed that a higher yield of lovastatin was obtained using pure sugar.

**Table 2:** Kinetic parameters of lovastatin production by *A. terreus* KPR12 grown in SWW

Kinetic parameters	Values
Lovastatin to biomass yield coefficient (Y_LOV/X_)	0.153 g g^−1^
Lovastatin to starch yield coefficient (Y_LOV/S_)	0.043 g g^−1^
Maximum specific formation rate of lovastatin (Q_max_)	0.0011 g g^−1^ h^−1^
Biomass to starch yield coefficient (Y_X/S_)	0.278 g g^−1^
Final biomass weight (X_Final_)	3.20 g L^−1^

In the current study, the biomass to starch yield coefficient and the maximum specific lovastatin formation rates (Q_max_) in SWW were 0.278 g g^−1^ and 0.0011 g g^−1^ h^−1^, respectively. Pawlak and Bizukojć [55] reported that biomass to lactose and biomass to glycerol yield coefficients by *A. terreus* were 0.55 g biomass/g lactose and 0.55 g biomass/g glycerol in fed-batch fermentation with an initial lactose and glycerol concentrations of 10 and 5 g L^−1^, respectively. The maximum specific lovastatin formation rate (Q_max_) was 0.00178 g g^−1^ h^−1^. Based on the kinetic results obtained in the present study, SWW can be used as the growth substrate for the effective production of various biomolecules. Moreover, the synthesis of secondary molecules depends on strains and culture conditions.

The lovastatin yield of *A. terreus* KPR12 under submerged fermentation in SWW was compared with other studies using diverse carbon sources (Table 3). In a study, Jaivel and Marimuthu [56] demonstrated that glucose was used as a sole carbon source to evaluate the ability of 10 fungal strains from various natural sources for the production of lovastatin and identified *A. terreus* (JPM3) as a better producer of lovastatin with a yield of 138.4 mg L^−1^. In our study, the fungal strain *A. terreus* KPR12 produced nearly 3.3 fold higher yield than the previous report [56]. Jia et al. [57] used soluble starch as a source of carbon and reported a 0.8 fold increase in the yield compared to the present study. Pecyna and Bizukojc [58] analyzed specific lovastatin yield during SmF using the lactose-to-glycerol ratio and found a lovastatin yield of 161.8 mg L^−1^. However, the current study indicates a 2.7 fold higher lovastatin output than the above studies. Sridevi and Charya [59] isolated various strains of *A. terreus* from soil samples and screened for the production of lovastatin using the agar plug assay method, and the maximum production of lovastatin (360 mg L^−1^) was obtained using *A. terreus* KSVL-SUCP-75. When compared to this value (360 mg L^−1^), the yield obtained from our research was 1.25 fold higher. In other studies, strain improvement techniques were adopted [60, 61] or supplements were added to the culture medium [31, 62] to increase the lovastatin titer. The results of the present study demonstrated that *A. terreus* KPR12 can be a potential lovastatin-producing strain, which effectively utilizes a waste stream to produce therapeutic metabolites.

**Table 3:** Lovastatin production by *A. terreus* KPR12 compared with other reports

*A. terreus* strain	Carbon source	Specific supplements/factors	Yield (mg L^−1^)	References
ATCC 20542	Lactose, glycerol	–	161.8	Bizukojc and Pecyna [80]
JPM3	Glucose	–	138.4	Jaivel and Marimuthu [56]
Z15-7	Glycerol	Mutant	916.7	Li et al. [61]
LA414	Soluble starch	Polyketide antibiotic	952.7	Jia et al. [62]
NRRL 255	Glucose malt extract milk powder	Reactor	920	Gupta et al. [81]
GD13	Lactose	Cyclic mutagenesis	1242	Kaur et al. [60]
LA414	Soluble starch	–	523.9	Jia et al. [57]
MUCL 38669	Lactose, glucose	Linoleic acid supplements	212.5	Sorrentino et al. [31]
KPR12	Starch-based SWW	–	450.79 (kinetic study)	Present study
*Monascus* strain				
MTCC 369	Glucose	–	737	Ahmad et al. [82]
MTCC 369	Glucose	–	351	Sayyad et al. [83]

### Yeast growth inhibition bioassay

*Aspergillus* species have proven to be a prolific source of secondary metabolites with interesting biological activities, including antibacterial activity [63, 64]. Lovastatin is known for its antifungal activity; it inhibits the growth of several fungal genera, including *Saccharomyces cerevisiae*, *Candida* spp., *Aspergillus* spp., and *Cryptococcus* spp., by inhibiting HMG-CoA reductase that depletes ergosterol, the fungal counterpart of cholesterol [65–67]. Both ergosterol and cholesterol are important for cell viability and membrane fluidity, and they follow a similar mechanism. The ethyl acetate extract of *Aspergillus* contains several antimicrobial compounds such as helvolic acid, monomethylsulochrin, ergosterol, terreic acid, butyrolactone, tensyuic acids, emodin, kojic acid, fumigaclavine, pseurotin, oleic acid, and *n*-hexadecanoic acid, in addition to lovastatin [68–71].

**Fig. 7:**
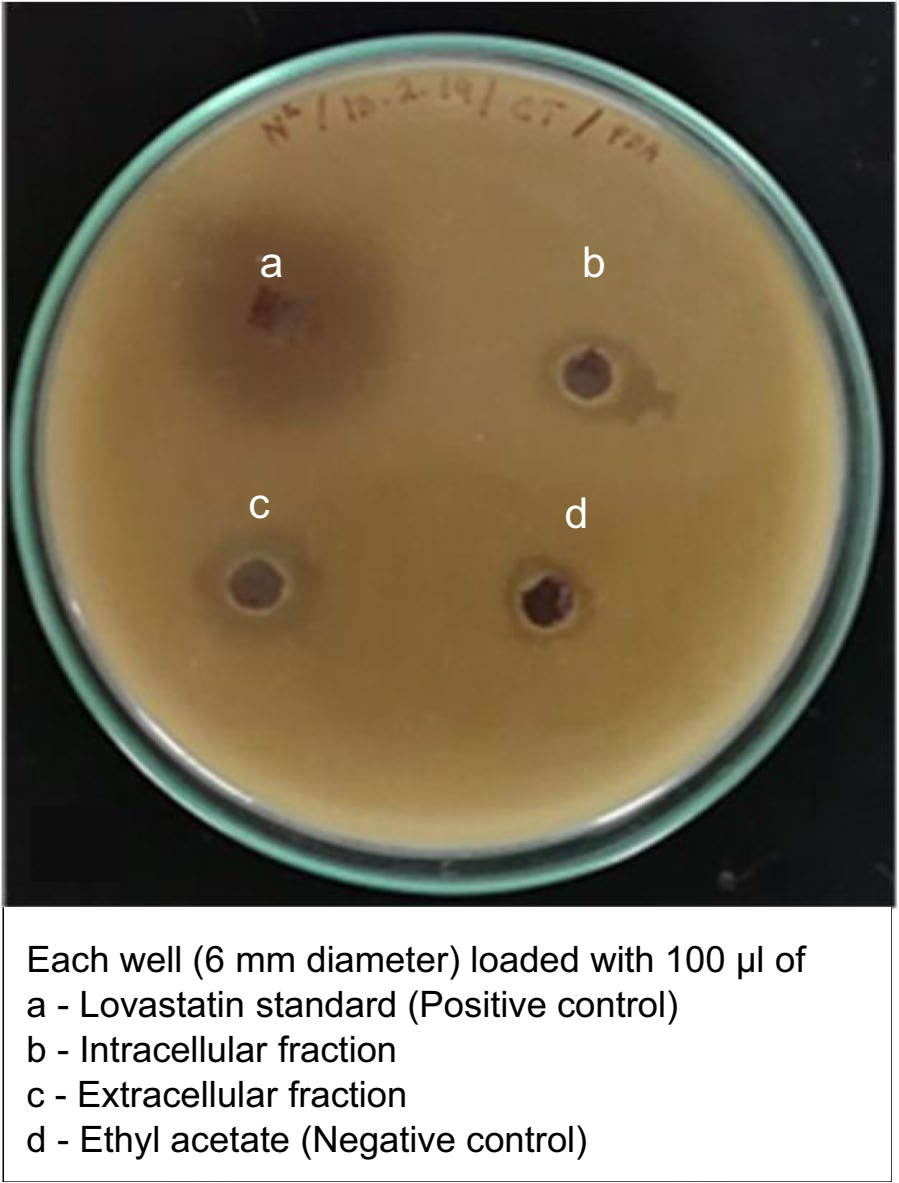
Lovastatin extract of *A. terreus* KPR12 inhibiting the growth of yeast observed by the zone of inhibition around a colony

In the present study, a yeast growth inhibition bioassay was performed to verify the antifungal potential of intra- and extracellular fractions of *A. terreus* KPR12 against *Candida tropicalis* ASY2. The growth of *C. tropicalis* ASY2 was inhibited in both control and fungal extracts, and clearing zones were observed (Fig. 7). The diameters of inhibition zones for both intra- and extracellular fractions in SWW were 12 and 14 mm, respectively. The clear zone may be due to the combined effects of lovastatin and unidentified antimicrobial compounds present in the fungal extracts.

### Simultaneous decontamination of SWW

**Fig. 8:**
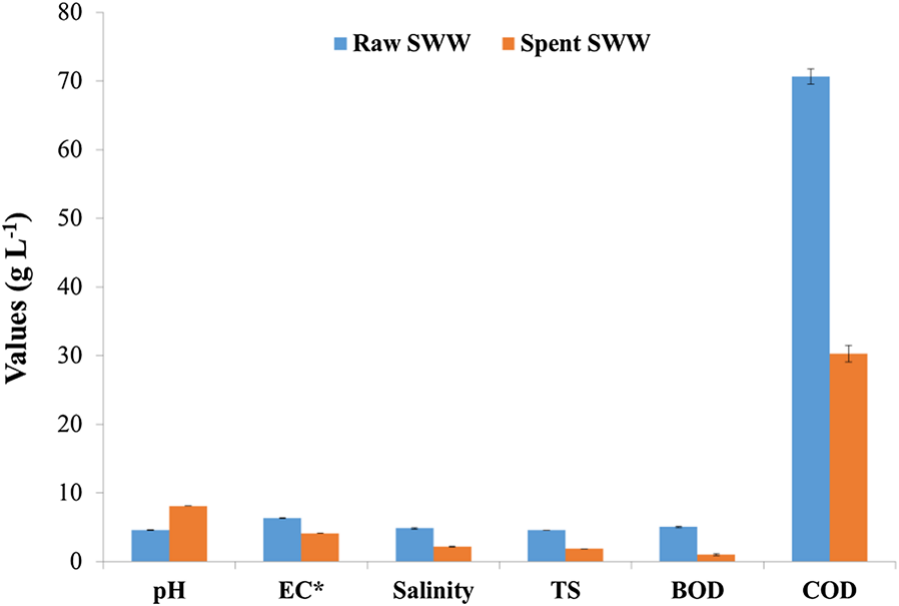
Decontamination of SWW during lovastatin production by *A. terreus* KPR12. EC, electrical conductivity; TS, total solids; BOD, biochemical/biological oxygen demand; COD, chemical oxygen demand. * dS m^−1^; units for all the other parameters are g L^−1^

In addition to lovastatin production, SWW can be treated and reused in the same industry for tuber washing or irrigation, or recreation purposes. The comparative evaluation of raw and spent SWW with national standards is presented in Table 4. We detected a slight increase in pH from 6.5 to 7.1 of the treated SWW, suggesting that alkalinization may be due to the secretion of ammonia and its related compounds by the fungus during its growth in SWW [72] (Fig. 8). The very high EC (6.2 dS m^−1^) of raw SWW was reduced (4.1 dS m^−1^) after the fermentation due to the soluble salts metabolized by the growth of fungi. The salinity, TS, and TDS contents were also reduced in SWW (Table 4). The high organic matter content in SWW (COD and BOD) could be effectively fermented by oleaginous fungi by oxygen consumption, and the level reduced to 30.27 and 1.03 g L^−1^, respectively. Our earlier report by *Candida tropicalis* ASY2 also supported the present investigation [28]. Almost all nitrogen content in the SWW was used as a nitrogen source for fungal growth. A small amount of phosphate (72.1 mg L^−1^) was available in the treated SWW. The bound cyanide in the tapioca roots was hydrolyzed by linamerase during the starch extraction process and left free cyanide in the waste stream. The microbe can grow and use cyanide-containing substrates through anaerobic metabolism, respiratory chain metabolism, and their ability to detoxify cyanide by splitting the CN radical into carbon and nitrogen [73, 74]. In the present study, the cyanide content was reduced to 1.54 mg L^−1^. The results were supported by preliminary works of Kandasamy [75], who isolated bacterial isolates that could tolerate up to 5 mM cyanide. However, further secondary treatment such as anaerobic digestion [76, 77] and extended aeration [78, 79] will further reduce the pollutant content of SWW and would pave the way for the reuse of spent SWW for various applications.

**Table 4:** Parametric comparison of raw and spent SWW with national standards

Properties	Raw SWW parameters estimated in our study	Treated SWW parameters estimated in our study	Different parameters of SWW reported in other studies^a^	National effluent standards for sago and starch industry
pH	4.67 ± 0.03	8.1 ± 0.02	4.5–5.5	6.5–8.5
EC (dS m^−1^)	6.3 ± 0.04	4.11 ± 0.0	1.7–3.3	–
Salinity (g L^−1^)	4.86 ± 0.09	2.15 ± 0.03	–	–
Total solids (g L^−1^)	4.57 ± 0.01	1.86 ± 0.01	0.8–12.45	0.1
Total dissolved solids (g L^−1^)	4.16 ± 0.02	1.32 ± 0.04	1.5–3.7	–
Starch (g L^−1^)	10.00 ± 0.07	0.002 ± 0.0	4–7	–
BOD (g L^−1^)	5.04 ± 0.08	1.03 ± 0.12	6.2–23.1	0.03
COD (g L^−1^)	70.22 ± 1.1	30.27 ± 1.2	11.08–19.08	0.25
NO_3_ (mg L^−1^)	3.10 ± 0.02	ND	–	10
NH_4_ (mg L^−1^)	5.48 ± 0.05	ND	–	50
PO_4_ (mg L^−1^)	611.67 ± 0.01	72.1 ± 0.04	–	5
Cyanide (mg L^−1^)	4.46 ± 0.02	1.54 ± 0.32	3.5–5.3	0.2

^a^Adopted from Sujatha and Kumar [74]; Bhaskar and Prasada Rao [84], and Priya et al. [85]

## Conclusion

*A. terreus* KPR12 produced an optimal titer of 450.79 mg L^−1^ lovastatin in SWW without additional nutritional input or strain improvement techniques. These findings pave the way for the cost-effective and efficient production of lovastatin by microbial fermentation, in which soluble starch in SWW is effectively converted into valuable by-products. Such an integrated application of *A. terreus* KPR12, along with the use of industrial waste streams, can provide new leads for the development of statin as well as effective waste management.

## Data Availability

All data generated or analyzed during this study are included in this published article.

## Abbreviations

SWW: Sago processing wastewater
SM: Synthetic medium
SmF: Submerged fermentation
SSF: Solid-state fermentation
EC: Electrical conductivity
BOD: Biochemical/biological oxygen demand
COD: Chemical oxygen demand
UV: Ultraviolet
FTIR: Fourier transform-infrared
HPLC: High-performance liquid chromatography
ANOVA: Analysis of variance
Y_LOV/X_: Lovastatin to biomass yield coefficient
Y_LOV/S_: Lovastatin to substrate yield coefficient
Y_X/S_: Biomass to starch yield coefficient
X_FINAL_: Biomass weight
Q_max_: Lovastatin specific formation rate
